# Temporally Correlated Deep Learning-Based Horizontal Wind-Speed Prediction

**DOI:** 10.3390/s24196254

**Published:** 2024-09-27

**Authors:** Lintong Li, Jose Escribano-Macias, Mingwei Zhang, Shenghao Fu, Mingyang Huang, Xiangmin Yang, Tianyu Zhao, Yuxiang Feng, Mireille Elhajj, Arnab Majumdar, Panagiotis Angeloudis, Washington Ochieng

**Affiliations:** 1 Centre for Transport Engineering and Modelling, Imperial College London, London SW7 2AZ, UK; lintong.li19@imperial.ac.uk (L.L.); m.huang18@imperial.ac.uk (M.H.); xiangmin.yang18@imperial.ac.uk (X.Y.); tz21@ic.ac.uk (T.Z.); y.feng19@imperial.ac.uk (Y.F.); a.majumdar@imperial.ac.uk (A.M.); p.angeloudis@imperial.ac.uk (P.A.); w.ochieng@imperial.ac.uk (W.O.); 2 State Key Laboratory of Air Traffic Management System, Nanjing 210007, China; 3 Astra-Terra Limited, London HA0 1HD, UK; m.el-hajj11@imperial.ac.uk

**Keywords:** horizontal wind-speed prediction, temporal correlation, quality indicator, LSTM, Bi-LSTM

## Abstract

Wind speed affects aviation performance, clean energy production, and other applications. By accurately predicting wind speed, operational delays and accidents can be avoided, while the efficiency of wind energy production can also be increased. This paper initially overviews the definition, characteristics, sensors capable of measuring the feature, and the relationship between this feature and wind speed for all Quality Indicators (QIs). Subsequently, the feature importance of each QI relevant to wind-speed prediction is assessed, and all QIs are employed to predict horizontal wind speed. In addition, we conduct a comparison between the performance of traditional point-wise machine learning models and temporally correlated deep learning ones. The results demonstrate that the Bidirectional Long Short-Term Memory (BiLSTM) neural network yielded the highest level of accuracy across three metrics. Additionally, the newly proposed set of QIs outperformed the previously utilised QIs to a significant degree.

## 1. Introduction

Wind speed is a fundamental meteorological attribute resulting from air passage from high pressure to low pressure areas. All living can sense the existence of wind, and wind speed also impacts all modes of transportation. Hence, studying wind speed is highly beneficial, as it significantly influences a diverse range of application domains. Moreover, by accurately predicting wind speed, we can effectively harness its benefits and mitigate its negative effects. The subsequent pair of illustrations exemplify the significance of accurately predicting wind speed in the realms of green and sustainable energy generation and aviation operation.

The world strives to construct a novel energy architecture, replacing traditional fossil energy, such as petroleum, coal, and natural gas, with green and renewable energy, such as wind and solar energy. In this way, the detrimental effects, such as the greenhouse effect and frequent earthquakes, can be alleviated, and the ecological environment can be more diverse and sustainable. According to [1], fossil and green energy account for approximately 83% and 13%, respectively. The world’s largest green and renewable energies are hydropower and wind energy, accounting for approximately 10% and 6%, respectively.

The U.S. Department of Energy anticipates increments of 120% and 183% of production for on- and off-shore wind energy in 2035 compared to 2019 [2]. Furthermore, this report highlights that future onshore wind turbines will be larger, with their location affecting the energy production rates and subsequent costs. Hence, accurate wind-speed prediction will result in improved site selection, windmill planning, and optimal wind machine size selection. This will lead to greater wind energy conversion.

Wind speed is critical in aircraft performance, particularly during takeoff, in-flight operations, and landing [3]. Crosswinds can make it difficult for pilots to maintain control of the aircraft during takeoff or landing, while wind shear can introduce sudden and hazardous changes in wind direction and speed, posing significant risks to flight safety [4]. Tailwinds and headwinds also affect runway distances and fuel efficiency, requiring pilots to adjust their flight strategies accordingly. In-flight, high wind speeds can lead to turbulence, microbursts, and gusts, increasing the potential for passenger injuries and posing risks to the aircraft’s structural integrity [5].

Statistics show that wind contributes to around 50% of weather-related aviation accidents in the United States [6]. One notable example is the 1999 crash of American Airlines Flight 1420, where severe crosswinds during landing at Little Rock National Airport, Arkansas, United States, caused the pilots to lose control, resulting in the aircraft overrunning the runway and crashing. Crosswind speed, in particular, affects not only airplanes but also drones, leading to flight delays, disturbances, and even accidents.

To mitigate these risks, aircraft must continuously adjust their trajectory based on current wind conditions to maintain their intended flight path, a phenomenon commonly referred to as the “wind effect”. Accurate wind-speed prediction is therefore essential to ensure the safety and security of aircraft, as sudden, unpredictable wind changes can render the aircraft uncontrollable.

The research on wind-speed prediction using Machine Learning (ML) models has been conducted for over two decades. Temporal or point-wise wind-speed data have been used for training and validating ML models. Initially, the seasonal or annual changes in wind speed were predicted at certain locations. Therefore, the case research may not necessarily apply to other scenarios. Mohandes et al. compared the performance of an Auto-Regressive (AR) model and Multi-Layer Perceptron (MLP) for wind-speed prediction in Saudi Arabia [7]. The MLP and AR results were 1.87 m/s and 2.88 m/s monthly, respectively, and 1.24 m/s and 1.37 m/s daily, respectively in Root Mean Square Error (RMSE). This explains that with more trainable parameters, the performance of MLP is better than AR in predicting wind speed accurately. Furthermore, as the model order increased, so did the accuracy of wind-speed prediction [2]. However, a high-order ML model could lead to overfitting.

A Seasonal Auto-Regression Integrated Moving Average and Least Square Support Vector Machine (SARIMA-LSSVM) for analysing and modelling wind speed was proposed by Guo et al. [8]. This hybrid model improved the multi-circumstance adaption compared to LSSVM, SARIMA, and ARIMA. All models mentioned above learned the law of monthly and annual wind-speed changes and cannot predict wind speed in real time. When facing a sudden change in wind speed within a very short period, wind energy production facilities cannot be protected, and aircraft cannot be adjusted in time, which leads to unpredicted accidents or losses.

ML regressive models with instantaneous or mean wind speeds differ because the predicted instantaneous data are not smooth. Outliers should also be predicted or classified accurately. As a transition, Erdem and Shi [9] implemented conventional, component-based, linked, and vector Auto-Regressive Moving Average (AR-MA) approaches for hourly mean wind-speed prediction. In contrast to the previous monthly or annual mean values, the hourly mean values can irregularly increase the wind speed from 2 m/s to 20 m/s in one day. Vector ARIMA performed best among these four models since the wind-speed directions were also considered. Predicting wind speed in both directions is more accurate than just predicting the magnitude of the wind speed. Before Recurrent Neural Networks (RNNs) were used on a large scale, most researchers used the ARIMA model and its variants, such as fractional-ARIMA [10] and Empirical Mode Decomposition (EMD) recursive-ARIMA [11] and hybrid models such as ARIMAANN [12] and ARIMA-Kalman [13], because of their simplicity and accurate prediction results when predicting original or differential time series.

Based on the nonlinearity, irregularity, randomness, and high complexity of the actual time-series data, increasing the order of the ARIMA model makes it difficult to achieve high-accuracy predictions and leads to overfitting. Furthermore, point-wise machine learning methods such as Support Vector Machine (SVM) and Decision Tree (DT) need more efficient handling of sequence relationships between input variables. Therefore, RNNs are often regarded as the most efficient method for time-series forecasting. RNNs are artificial neural networks, and the internal state of the network can exhibit dynamic time-series behaviour. However, with the increase in the duration of processing time series, the problem of gradient vanishing often occurs during training RNNs using conventional activation functions, which limits the prediction accuracy of RNNs. Hence, LSTM is based on the RNNs, adding multi-threshold gates to solve storage and forgetting problems [14]. There are also other variants of RNN models, which are described in detail in Section 2.

Zhou et al. [15] proposed a novel surrogate model for predicting wind turbine wakes, termed DPLWP, which integrates delayed proper orthogonal decomposition (d-POD) with LSTM networks with high-fidelity computational fluid dynamics (CFD) data. The d-POD method effectively reduces the dimensionality of high-dimensional data, allowing for the extraction of significant modes and coefficients that capture the intrinsic features of the wake flow. This study demonstrated that the low-order d-POD modes represent large-scale coherent structures in the wake, and the introduction of d-POD significantly reduces the prediction error of the LSTM network.

Duan et al. [16] analysed Back Propagation Neural Networks (BPNNs), Convolutional Neural Networks (CNNs), RNN, Gated Recurrent Unit (GRU), Linear Regression Networks (LRNs), LSTM, and their hybrid models. Experimental results showed that the GRU performed best in Mean Absolute Error (MAE) and Root Mean Square Error (RMSE). Moreover, Yu et al. [17] proposed a CNN-Time Frequency Recurrent Network (TFR) to learn hidden time-frequency features. Here, CNN-LSTM, CNN, GRU, and CNN-TFR performed best among all models with MAE less than 0.70 m/s and RMSE less than 0.90 m/s. The input for their work is statistical wind-speed data, including maximum, minimum, mean, and standard deviation.

Liang et al. [18] proved Bidirectional (Bi-LSTM) could generate the most accurate and stable wind-speed prediction results compared to the GRU, LSTM, BP, Radial Basis Function (RBF), and Wavelet Neural Network (WNN) in both theoretical and experimental ways. In addition, other hybrid models, including Ensemble Empirical Mode Decomposition (EEMD), Genetic Algorithm (GA)-LSTM [19], Wavelet Decomposition (WT)-Bi-LSTM [20], and Particle Swarm Optimisation (PSO)-Bi-LSTM [21], are also used to improve the prediction accuracy and other metrics.

Bilgili et al. found that the performance of the ANN model was affected by geographic information [22]. Different geographical locations have different weather and landforms. Consequently, the value range and degree of change in wind speed also differ. Therefore, machine learning models using only historical wind-speed data as input cannot predict the wind speed in real time well. However, suppose other characteristics such as temperature, humidity, and air pressure are also fed into models. In that case, the current weather and altitude can be estimated, and the wind speed can be predicted accurately. For design purposes, performance analysis, and running cost estimation of renewable energy systems, variations in meteorological parameters such as wind speed, humidity, temperature, and solar radiation are also required [23].

Based on the discussion above, this paper initially overviews the definition and characteristics of more Quality Indicators (QIs) relevant to the wind-speed prediction task other than wind speed itself, such as humidity, temperature, and solar radiation. The sensors capable of measuring these QIs will also be introduced, and the feature importance of these QIs will be analysed. Then, a new set of QIs will be proposed to feed into machine learning models to improve the accuracy of wind-speed prediction. The performance of temporally correlated deep learning models, including LSTM, GRU, CNN-LSTM, and Bi-LSTM, and point-wise machine learning models, will be introduced and compared. The results of wind-speed prediction will be provided and analysed finally.

## 2. Machine Learning Models for Wind-Speed Prediction

Over the past two decades, numerous traditional machine learning models and temporally correlated deep learning models have been used for wind-speed prediction [24,25]. This section introduces the forecasting models utilised in the experimentation. The initial three models are classified as point-wise machine learning models, whereas the last five are categorised as temporally correlated models. The point-wise machine learning model exclusively utilises the current data as input, but the temporally correlated model incorporates both the current and previous data.

### 2.1. Polynomial Regression

Polynomial regression is a regression in which the relationship between QIs and projected values is modelled as an nth-degree polynomial. This paper employs a polynomial regression model of the third degree, which is

(1) $$y=\alpha_{0}x+\alpha_{1}x^{2}+\alpha_{2}x^{3}+\varepsilon$$

where *x* is a vector of QIs, *y* is the predicted wind speed, and $\alpha_{0}$, $\alpha_{1}$, $\alpha_{2}$ are the trained coefficients. This algorithm is sensitive to outliers, so one or two can hurt its performance. Tarade and Katti [26] predicted wind speed using temperature and pressure. The wind speed ranged from 0 to 8 m/s, and the RMSE of the predicted value was 2.200 m/s. The accuracy of the machine learning model is not optimal due to the absence of temporal correlation.

### 2.2. Random Forest

Random forest is an ensemble-learning method for regression. In statistics and machine learning, the ensemble-learning method employs multiple learning algorithms to achieve superior predictive performance than any original learning algorithms alone. The output of a random forest is the average of the individual Decision Trees’ outputs. The random forest model is significantly more effective than a single Decision Tree at avoiding overfitting. Moreover, the random forest generally outperforms a single Decision Tree. For instance, Vassallo et al. [27] determined the RMSE of the predicted wind speed to be 0.729 m/s, which is superior to the polynomial regression model.

### 2.3. Multilayer Perceptron

**Figure 1:**
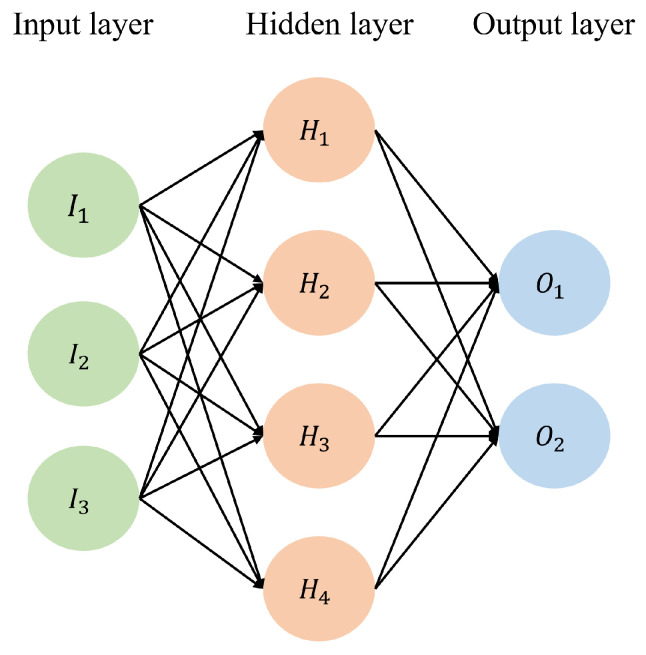
Structure of MLP.

The Artificial Neural Network (ANN) is a computational network inspired by biology. It simulates a biological network utilising simplified concepts of biological neural systems. Non-deep neural networks frequently use Multilayer Perceptron (MLP) with a back-propagation learning algorithm for classification and regression. Three types of layers exist: input, hidden, and output. The illustration of MLP’s structure appears in Figure 1. The MLP is capable of handling non-linear regression problems and massive input data. However, the training time of the MLP is quite long.

### 2.4. ARIMA

ARIMA denotes AutoRegressive Integrated Moving Average. ARIMA is frequently used to analyse and forecast stationary time series as a general model. Through differentiation, the non-stationary time series can be transformed into stationary. A random time-series variable is stationary if its statistical properties remain constant over time. This model consists of three components:

1. AutoRegression: In an autoregression model, the forecast for the target variable is derived from a linear combination of its past values. AutoRegression refers to the regression of a variable against itself. Thus, a p-order autoregressive model can be expressed as
  (2) $$y_{t}=c+\sum_{i=1}^{p}\phi_{t-i}y_{t-i}+\varepsilon_{t}$$
  where $\varepsilon_{t}$ represents white noise. This is similar to multiple regression, but predictors have lagged $y_{t}$ values. This is known as an AR(p) model, an autoregressive order p model.
2. Integrated: Differentiation can assist in stabilising the mean of a time series by eliminating level changes, thereby eliminating or reducing trend and seasonality.
3. Moving Average: Instead of using past values of the predicted variable in a regression, a moving average model uses past predicted errors in a model that is similar to a regression,
  (3) $$y_{t}=b+\sum_{j=1}^{p}\theta_{j}y_{j}+\varepsilon_{t}$$

The ARIMA model is obtained by combining differencing with autoregression and a moving average model. For example, Kavasseri and Seetharaman [10] predicted the wind speed with both ARIMA and fractional-ARIMA models with RMSE 11.87 and 5.25 m/s. Moreover, Liu et al. [13] used two hybrid ARIMA models to predict wind speed with RMSE 0.624 and 0.717 m/s.

(4) $$y_{t}=b+\sum_{i=1}^{p}\phi_{i}y_{i}+\sum_{j=1}^{p}\theta_{j}y_{j}+\varepsilon_{t}$$

### 2.5. LSTM

**Figure 2:**
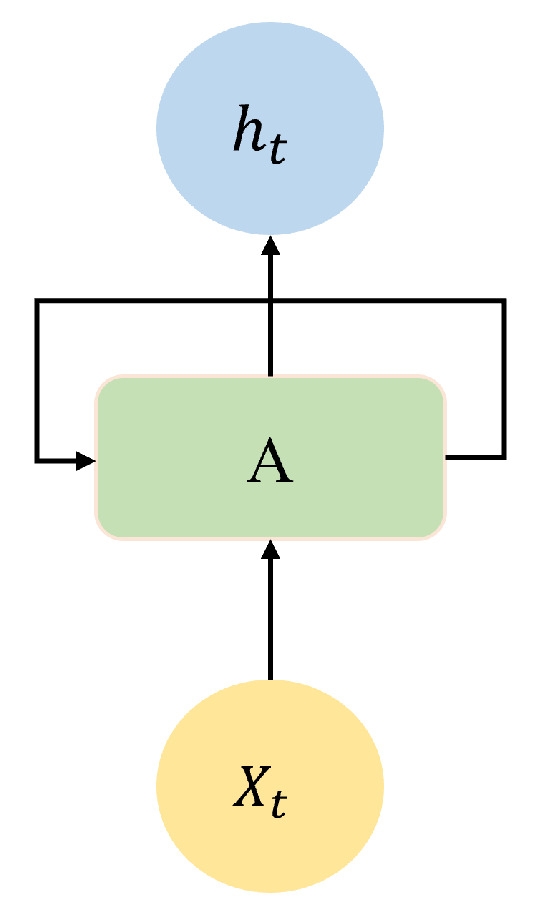
Structure of an RNN cell.

Unlike general regression, time-series regression uses information from past epochs to predict current values [28]. A feedforward neural network with internal memory is generalised by a Recurrent Neural Network (RNN). RNN is recurrent because it performs the same function for each data input, while the output of the current input is dependent on the previous computation. After the output has been generated, it is duplicated and sent back into the recurrent network. For decision-making, it considers the current input and the output learned from the past input (see Figure 2).

**Figure 3:**
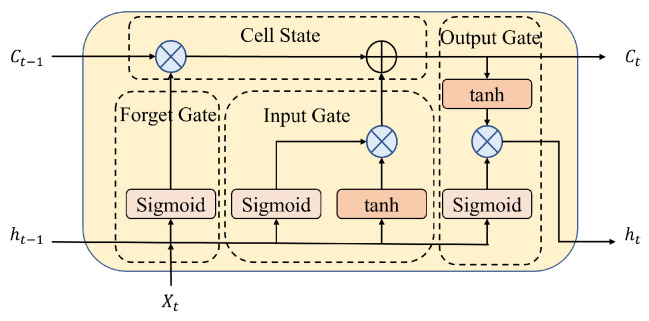
Structure of an LSTM cell.

Unlike feedforward neural networks, RNNs can process time-series inputs using their internal state (memory). Therefore, all inputs are interconnected within the RNN. The primary advantage of the RNN is that it can model time-series data so that each sample can be assumed to be dependent on the past ones. However, the gradient vanishing and exploding issue cannot be circumvented. The LSTM model was proposed to address this issue [26,27]. Adding three gates (input, forget, and output) allows past data usage to be controlled. Figure 3 illustrates the LSTM model used in our experiments.

The input gate serves as the cell state input. A sigmoid function is first applied to the past hidden state and current input to determine which values will be updated. Subsequently, the network is regulated by sending the same two inputs to the tanh activation. Finally, multiply the tanh output by the sigmoid output to determine which data are essential for updating the cell’s state.

The output of the forget gate is multiplied pointwise with the input from the past cell state. If the forget output is 0, the output of the previous cell is discarded. This output is added to the input gate’s output to update the cell’s state. For example, the cell’s current state will be the input for the following LSTM unit.

The hidden state contains information about past inputs and is used for prediction. The output gate controls the concealed state in the current epoch. The past hidden state and current input are passed through the sigmoid function. This output is multiplied by the output of the tanh function to determine the hidden state. The outputs of a conventional LSTM unit are the current state and the current hidden state.

Dropout is a regularisation technique that prevents neural network overfitting. Regularisation strategies such as L1 and L2 reduce overfitting by modifying the cost function. However, dropout modifies the network itself. During each training, it eliminates neurons from the neural network at random.

### 2.6. GRU

GRU is an acronym for Gated Recurrent Unit [29]. It consists of two gates: the update gate and the reset gate. The update gate determines how much historical data must pass to the subsequent state. Thus, the vanishing gradient problem is resolved. The model uses the reset gate to determine whether or not past information is crucial. If not, this information should be disregarded.

**Figure 4:**
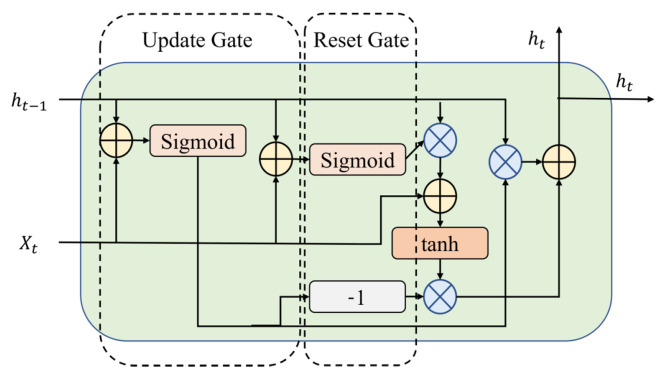
Structure of a GRU cell.

Unlike the three-gate LSTM, the two-gate GRU consumes less memory and outputs results faster. However, the LSTM typically produces more accurate predictions because more parameters are trained within the model. See Figure 4.

### 2.7. CNN-LSTN

CNN-LSTM combines CNN layers for feature extraction on input data and LSTMs for time-series prediction [30]. CNN layers are effective for time-series applications because of the convolution kernel’s ability to extract unobvious data features in the time direction. The LSTM architecture uses CNN’s extracted features as the input. For the LSTM model to discover the connections within the input and output, the training data and various LSTM network gates are continuously modified.

**Figure 5:**
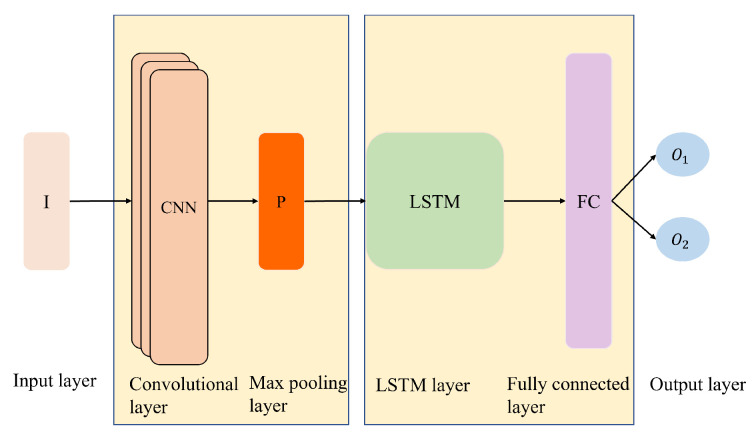
Structure of CNN-LSTM.

In contrast to other deep learning models, such as LSTM and CNN, the CNN-LSTM model will not explicitly produce a vector series. Instead, the model will consist of two parts: an encoder model that reads and encodes the input sequence and a decoder model that reads the encoded input sequence and makes a single-step prediction for each element in the output sequence. Figure 5 depicts the structure of the CNN-LSTM model employed in this study.

### 2.8. Bi-LSTM

**Figure 6:**
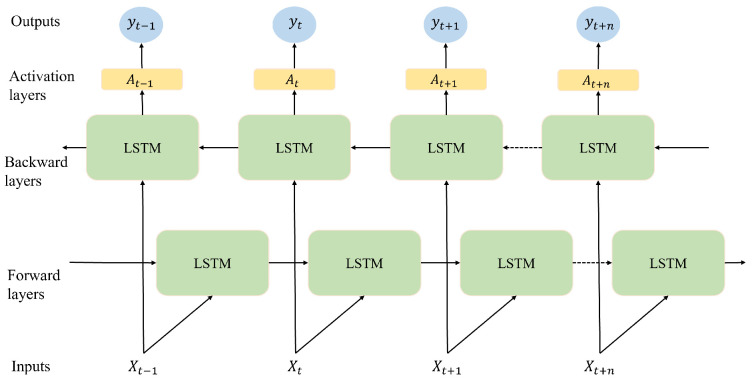
Structure of Bi-LSTM.

The input flows in both directions in the Bi-LSTM model, which is an extension of the unidirectional model (see Figure 6) [31]. The LSTM model is applied to the input time series in the first round. In the second round, it is fed the input time series in reverse form. The outputs of both LSTM layers are then combined by averaging, summing, multiplying, or concatenating them. This way, learning to automatically extract unobvious data features in the temporal direction can be improved [32].

In summary, LSTM and GRU are advanced, recurrent neural networks designed to handle long-term dependencies in sequential data. LSTM uses three gates (input, forget, and output) to control the flow of information, making it effective for complex time-series data but computationally heavier. Conversely, GRU simplifies the architecture by using only two gates (reset and update), making it faster and less prone to overfitting but slightly less powerful for some tasks. CNN-LSTM combines convolutional layers for feature extraction with LSTM layers for sequence prediction. Bi-LSTM extends LSTM by processing data in both forward and backward directions, allowing it to capture more context from sequential data, and enhancing performance in tasks.

## 3. Quality Indicators and Sensors

**Figure 7:**
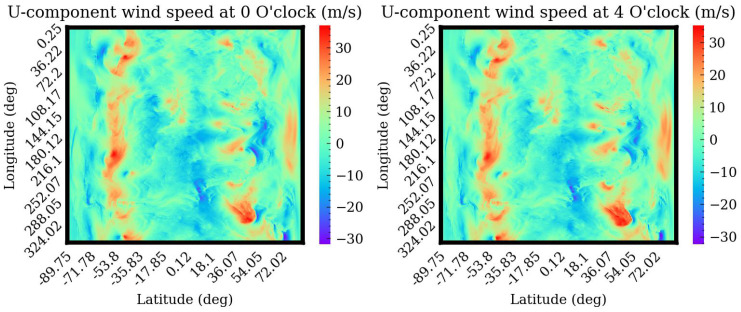

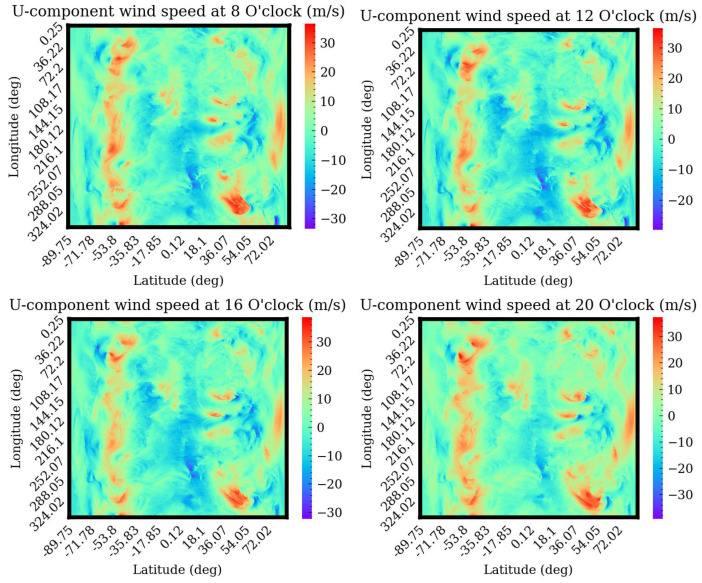
U-component wind speed on 6 January 2015.

**Figure 8:**
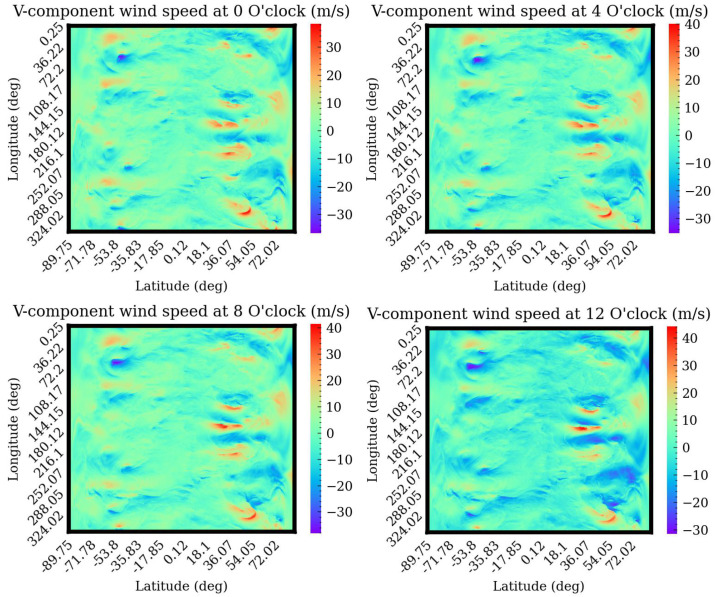

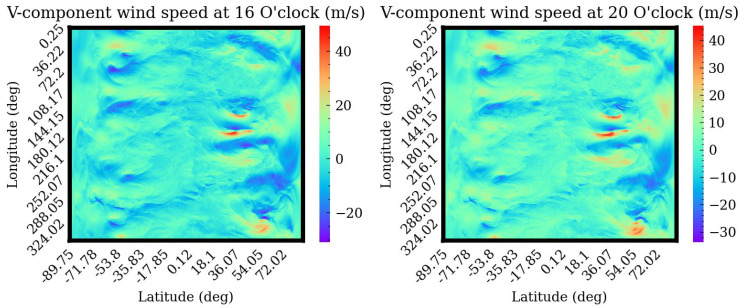
V-component wind speed on 6 January 2015.

Horizontal wind speed is often described in two dimensions: direction and magnitude. It can be divided into east–west (U) and north–south (V) components and predicts the magnitude of wind speed in these two directions. Figure 7 and Figure 8 depict the wind speed from 2 a.m. to 8 p.m. on 1 June 2015. From the figure, the wind speed changes slowly with time. Therefore, the temporal correlation of wind speed is considered in this paper. Since machine learning algorithms are used to predict wind speed, related Quality Indicators (QIs) and relevant sensors should be introduced and studied. The list of QIs are

1. Divergence;
2. Fraction of cloud cover;
3. Geopotential;
4. Ozone mass mixing ratio;
5. Potential vorticity;
6. Relative humidity;
7. Relative vorticity;
8. Specific cloud ice water content;
9. Specific cloud liquid water content;
10. Specific humidity;
11. Specific cloud rain water content;
12. Specific cloud snow water content;
13. Temperature;
14. Vertical velocity.

### 3.1. Divergence

The horizontal divergence is the rate per square metre at which air spreads horizontally from a point [33]. Divergence happens when air streams flow in opposing directions or when a stronger wind moves away from a weaker wind. Rising air results from divergence in the upper regions of the atmosphere. The air’s velocity of ascent is influenced by other atmospheric lifting or sinking forces as well as the size of the divergence. It is related to vertical motion and variations in local pressure through continuity equations and equations of motion. This QI is positive when air expands or diverges and negative when air converges or concentrates.

Doppler radars include Extended Velocity-Azimuth Display (EVAD), Concurrent Extended Velocity-Azimuth Display (CEVAD), Volume Velocity Processing (VVP), Light Detection and Ranging (LIDAR), radar wind profilers and mesonets, as well as weather satellites with tropospheric monitor; reflectometers or scatterometers can be used to determine the horizontal divergence [34,35,36]. Doppler radars provide accurate information on wind direction and velocity at different elevations by measuring the velocity of precipitation particles along the radar beam. LIDAR systems yield high-resolution data for divergence calculations by detecting aerosol particle mobility. A wind profiler equipped with sound waves and radar (SODAR) at different heights can be used to calculate divergence. Additionally, a high-density mesonet has several profilers inside. Moreover, by following the movements of clouds and water vapour, geostationary and polar-orbiting weather satellites equipped with atmospheric motion vector algorithms make it possible to derive horizontal wind fields and divergence across large regions. Additionally, the roughness of the ocean’s surface can be analysed by satellite reflectometry, and from this roughness, wind direction and velocity can be calculated. The backscatter of microwave signals that the satellite emits and that are reflected from the ocean surface is measured by scatterometry. Estimating wind vectors using many scatterometer data at various angles helps increase accuracy.

### 3.2. Fraction of Cloud Cover

A cloud fraction of one indicates that the pixel is completely covered with clouds (liquid or ice), whereas a zero fraction indicates that the pixel is cloud free. This QI is correlated with sunshine duration, with the least cloudy regions being the sunniest and the cloudiest being the least sunny. Zainab et al. [37] demonstrated a negative correlation between wind speed and cloud fraction. Therefore, reduced cloud cover will increase wind speed. Shane and Terry L [38] also proved that the cloud cover fraction indirectly affects temperature, relative humanity, and wind speed.

(5) $$\begin{array}{cc} T & -\left(0.55-\left(0.55\times \frac{RH}{100}\right)\right)\times \left(T-58\right)\\ \; & =0.0926CC+79.7\\ \; & =-1.072WS+88.572 \end{array}$$

where *T*, CC, RH, and WS are temperature, cloud cover, relative humanity, and wind speed, respectively.

Various sensors used to quantify cloud coverage include ceilometers, Cloud-Aerosol LIDAR and Infrared Pathfinder Satellite Observations (CALIPSO), cloud radars, Geostationary Operational Environmental Satellite (GOES), Moderate Resolution Imaging Spectroradiometer (MODIS), pyranometers and pyrheliometers, sky imagers, and Visible Infrared Imaging Radiometer Suite (VIIRS) [39,40,41,42]. Celiometers are instruments that utilise LIRAD technology to measure the height of cloud bases. Additionally, they can be employed to estimate the proportion of the sky covered by clouds by sensing their presence above. CALIPSO employs LIDAR technology to acquire vertical profiles of clouds and aerosols, providing precise data on cloud percentage, particularly for thin clouds or with several layers. Geostationary Operational Environmental Satellites (GOESs) offer uninterrupted surveillance of cloud cover by utilising infrared, visible, and water vapour channels to calculate cloud fraction. The SLSTR instrument functions by observing thermal infrared radiation radiating from the Earth’s surface, allowing for accurate measurements of sea and land surface temperatures affected by the amount of cloud cover. The Sentinel-3 satellite is equipped with the Sea and Land Surface Temperature Radiometer (SLSTR) and the Ocean and Land Colour Instrument (OLCI) to accurately observe and assess cloud coverage and percentage. OLCI or sky imagers collect photographs of the land or sky at fixed intervals. Image-processing algorithms are used to analyse these photos to calculate the cloud fraction. Pyranometers and pyrheliometers indirectly estimate cloud cover by measuring variations in sun radiation. VIIRS can capture high-resolution photos of the Earth’s surface using various spectral bands, enabling precise observation of cloud cover.

### 3.3. Geopotential

This QI refers to the gravitational potential energy of a unit mass about the mean sea level at a specific place. This picture illustrates the fluctuations in the geopotential height of the Earth’s surface. It is frequently called orography. The geopotential height and temperature are always employed in the empirical formula to calculate wind speed. For instance, the rise in wind speed and height were correlated using the Hellman power law [43]. Many organisations use the most popular wind profile model to calculate vertical wind increases across flat terrain.

(6) $$\frac{u}{u_{0}}={\left(\frac{H}{H_{0}}\right)}^{\alpha }$$

where *u* is wind speed at height *H*, and $u_{0}$ and $H_{0}$ are reference wind speed and height. α is an empirical coefficient that depends on the atmosphere is stability.

Geopotential measurement sensors can be classified into three primary categories: gravity based, GNSS signal based, and inertia based. Gravity-based sensors, including gravimeters, satellite altimeters, and satellite gravity missions like GRACE and GOCE, are used to monitor changes in the Earth’s gravity field to determine variances in geopotential. GNSS signal-based and inertia-based approaches involve using GNSS receivers, barometers, radio occultation devices, and inertial navigation systems. These methods utilise accurate location, atmospheric pressure, and motion data to determine geopotential heights [44].

### 3.4. Ozone Mass Mixing Ratio

The amount of ozone in one kilogram of air is known as the mass mixing ratio of ozone. Organisms that live on the surface are shielded from the harmful effects of ultraviolet (UV) light from the Sun by the naturally produced ozone in the stratosphere. Air movement also disperses ozone throughout the environment. The amount and direction of wind-speed impact the ozone mass mixing ratio, according to Seguel et al. [45]. The change in this QI is relatively minimal because of the low wind speed in winter. In contrast, this QI varies significantly in the other three seasons. Wind speed and the ozone mass mixing ratio change have a positive correlation.

Some of the sensors used to measure the mass mixing ratio of ozone include Brewer or Dobson spectrophotometers, chemiluminescence ozone analysers, Electrochemical Concentration Cell (ECC) ozonesondes, LIDAR, satellite-based instruments such as Global Ozone Monitoring Experiments (GOME), Microwave Limb Sounder (MLS), Ozone Monitoring Instrument (OMI), Total Ozone Mapping Spectrometer (TOMS), and UV photometers [46,47,48,49]. Spectrophotometers quantify the overall amount of ozone by monitoring the absorption of solar ultraviolet light at various wavelengths. These instruments are frequently utilised in terrestrial-monitoring stations and aerial platforms. Chemiluminescence ozone analysers detect ozone by quantifying the luminescent emission resulting from the chemical reaction between ozone and a particular reagent. ECC ozonesondes are airborne devices that assess ozone levels by detecting the electric current generated in an electrochemical cell. They offer vertical ozone profiles spanning the Earth’s surface to the stratosphere. Satellite-based equipment uses UV, visible, and microwave spectroscopy to accurately measure the ozone mass mixing ratio. This allows for the generation of comprehensive global and vertical profiles of ozone concentrations. LIDAR devices employ laser pulses to quantify the ozone levels at various elevations. Differential absorption refers to the phenomenon where the absorption of certain substances or wavelengths of light varies in different materials or environments. LIDAR (DIAL) is a specialised form of measurement that quantifies ozone levels by comparing signals reflected at two distinct wavelengths.

### 3.5. Potential Vorticity

The potential vorticity gauges how freely the air in the atmosphere can spin. It is used to pinpoint the locations where powerful windstorms are most likely to form and strengthen. Strong windstorms can form when a column of air in the atmosphere starts to rotate. The potential vorticity of an atmospheric column of air is determined using wind, temperature, and atmospheric pressure. Horizontal vorticity is characterised by a shift in wind direction or speed with height (the spin is about a horizontal axis). The spinning motions of air are known as vorticity. Furthermore, it is a vector whose elements concerning these three directions vary in a manner that is proportionate to the fluctuations of the wind’s transverse components in perpendicular directions.

The sensors used to measure potential and relative vorticity include Doppler weather radars, satellite-based equipment such as the Atmospheric Infrared Sounder (AIRS), Advanced Scatterometer (ASCAT), radio occultation sensors, and wind profilers. These sensors are similar to those used for measuring horizontal divergence. Oceanographic equipment, such as the Acoustic Doppler Current Profiler (ADCP) [50], utilises several sensors. Autonomous buoys collect data on temperature, salinity, and pressure in the ocean, which is crucial for calculating oceanic potential vorticity. An Acoustic Doppler Current Profiler (ADCP) is a device that uses the Doppler effect of sound waves to quantify water current velocities at various depths. It does this by analysing the scattering of sound waves from particles in the water column.

### 3.6. Relative Humidity

This QI shows the air saturation level as a percentage of the water vapour pressure. The water vapour starts to deposit as liquid water or ice or starts to condense. It is calculated for saturation over water at temperatures above 0 degrees Celsius (273.15 K). Calculations for saturation on ice are made at temperatures lower than −23 degrees Celsius. Using a quadratic function to interpolate the ice and water values, this parameter is determined between −23 °C and 0 °C. As Ravi and Paolo show, higher wind speeds correlate with lesser humidity and vice versa. (1) There is a strong correlation between wind speed and relative humidity. The rate of water evaporation is influenced by how quickly air passes across the water’s surface [51].

Humidity sensors encompass a range of technologies, including capacitive and resistive sensors, dew point sensors, gravimetric and thermo hygrometers, infrared gas analysers, LIDAR, microwave radiometers, psychrometers, radiosondes, as well as satellite-based sensors like the Atmospheric Infrared Sounder (AIRS), Advanced Microwave Sounding Unit (AMSU), and Moderate Resolution Imaging Spectroradiometer (MODIS) [52,53,54]. Humidity sensors quantify humidity levels by sensing variations in a hygroscopic substance’s capacitance or electrical resistance. Dew point hygrometers determine the temperature at which air reaches its saturation point. Thermo hygrometers utilise capacitive or resistance sensors to gauge temperature and relative humidity. They are frequently employed in meteorological stations and for monitoring indoor air quality. Infrared gas analysers quantify the level of water vapour in the atmosphere by detecting the absorption of infrared radiation at distinct wavelengths. LIDAR systems can quantify the amount of water vapour in the atmosphere by detecting the reflection of laser pulses. Radiosondes are sensors carried by balloons that measure the atmosphere’s temperature, pressure, and humidity as they rise vertically. Microwave radiometers quantify the magnitude of microwave radiation discharged by atmospheric water vapour. Satellite-based sensors utilise infrared and microwave radiometry to accurately monitor atmospheric temperature and humidity profiles. This equipment offers comprehensive worldwide data on atmospheric moisture, essential for weather prediction, climate monitoring, and atmospheric research.

### 3.7. Relative Vorticity

It gauges the horizontal rotation of air around a vertical axis about a fixed point on the surface of the Earth. On the scale of weather systems, ridges (weather features that bring calm or mild winds) are related to clockwise rotation. In contrast, troughs (weather features that can bring precipitation) are associated with anticlockwise rotation (in the northern hemisphere).

### 3.8. Specific Cloud Ice Water Content

The mass of cloud ice particles per kilogramme of the entire mass of wet air is shown by this QI. The combination of dry air, water vapour, cloud liquid, cloud ice, precipitation, and snowfall creates the total moist air mass. The primary value of a grid box is this QI. Water content can be used to visually represent wind speed in typhoon and tornado weather [55].

Sensors that can measure the content of ice, liquid, rain, and snow water include cloud radars, disdrometers, satellite-based sensors like AMSR, CALIPSO, CloudSat’s Cloud Profiling Radar (CPR), Global Precipitation Measurement (GPS), MODIS, and microwave radiometers and snow gauges [56,57]. Disdrometers quantify the amount of rainwater by examining the dimensions and speed of raindrops. Microwave radiometers quantify the total amount of liquid water in clouds and offer estimations of water vapour. Satellite-based instruments employ sophisticated technologies like radar, microwave radiometry, and LIDAR to offer precise vertical profiles and worldwide assessments of cloud ice, liquid, rain, and snow water content. These measurements are crucial for comprehending cloud characteristics, precipitation mechanisms, and atmospheric dynamics. Snow gauges determine the amount of water in snow by collecting and measuring the weight of the snowfall.

### 3.9. Specific Cloud Liquid Water Content

This QI shows the specific cloud liquid water content or the number of cloud liquid water droplets per kilogramme of the total moist air mass. The mean value of a grid box is also the QI. According to Lyzenga’s [58] experimental findings, the wind speed increases as liquid water content does. This lends more credence to the notion that water content and wind speed are inversely related.

### 3.10. Specific Humidity

Specific humidity is the quantity of water vapour per kilogramme of moist air. The difference between relative and specific humidity is that relative humidity, frequently expressed as a percentage, indicates the current state of absolute humidity with the maximum humidity possible at the same temperature. On the other hand, specific humidity is the ratio of water vapour mass to total moist air parcel mass.

### 3.11. Specific Cloud Rain Water Content

As precipitation, the mass of water produced by large-scale clouds of raindrop size can fall to the ground. Clouds contain a continuum of water droplets and ice particles of varying sizes. This QI is also the grid box’s mean value.

### 3.12. Specific Cloud Snow Water Content

The accumulation of snow-covered ice crystals can form large-scale precipitation that falls to the ground. The mean value of the grid box is also this QI. After an experiment, Herrero et al. [59] found that relative humidity and wind speed alter more noticeably when snow.

### 3.13. Temperature

This QI stands for the temperature of the atmosphere. To convert a Kelvin temperature to degrees Celsius, deduct 273.15 from the value. It is accepted that the association between temperature change and wind speed is substantial. Due to the wide temperature difference, there may be a strong wind. The QIs at this point and their temporal association must, therefore, be considered. Machine learning models with a temporal correlation will produce more precise predictions when chosen. Several empirical models describe wind speed using temperature and other QIs.

Temperature sensors quantify temperature in diverse settings and contexts [60]. Common temperature sensors include thermocouples, which produce voltage differences at the junction of two metals; Resistance Temperature Detectors (RTDs), which establish a relationship between resistance and temperature; and thermistors, which alter their resistance in response to temperature changes. Infrared sensors quantify temperature by sensing emitted infrared radiation, whereas semiconductor sensors utilise the voltage drop across a diode or the change in resistance in a semiconductor. Thermal cameras transform infrared radiation into visual representations that depict the temperature distribution. In contrast, digital temperature sensors offer measurements in a digital format and include supplementary capabilities such as data logging. These sensors play a vital role in industrial processes, scientific research, consumer electronics, and environmental monitoring.

### 3.14. Vertical Velocity

Vertical velocity refers to the speed at which air moves upwards or downwards. It can be utilised to understand the macroscopic movements of the atmosphere, such as upward and downward motion areas. The previously described sensors for velocity measurement have already been discussed. Table 1 provides a concise overview of the QIs and the corresponding sensor types.

## 4. Experimental Evaluation

This paper uses data from the European Centre for Medium-Range Weather Forecasts (ECMWF) Reanalysis v5 (ERA5). From 1950 to 2022, hourly estimates are available for many atmospheric, ocean-wave, and land-surface quantities. This dataset is a subset of the complete ERA5 dataset at its native resolution that has been gridded along latitude and longitude. Data were gridded onto a regular 0.25-degree latitude–longitude grid for the reanalysis and a 0.50-degree grid for uncertainty estimation. This dataset includes all QIs and the wind speed in two directions. The selected dates are 31 May and 1 June 2015, on which the weather was changeable. To analyse the temporally correlated characteristics, 29 consecutive hours of data were collected. The selected HPa values are 875, 900, 925, 950, 975, and 1000 based on the aircraft flight trajectory. To analyse the effect of the deep learning models at different latitudes and longitudes, four capital cities with large flight volumes. Beijing (39.917, 116.383), London (51.510, 359.882), Nairobi (−1.286, 36.817), and Washington DC (38.889, 282.949) were selected. With each capital city as the centre, data within 5 degrees of latitude and longitude were used for experiments. These four cities’ temperature, relative humidity, wind speed, and barometer changes are shown below.

**Figure 9:**
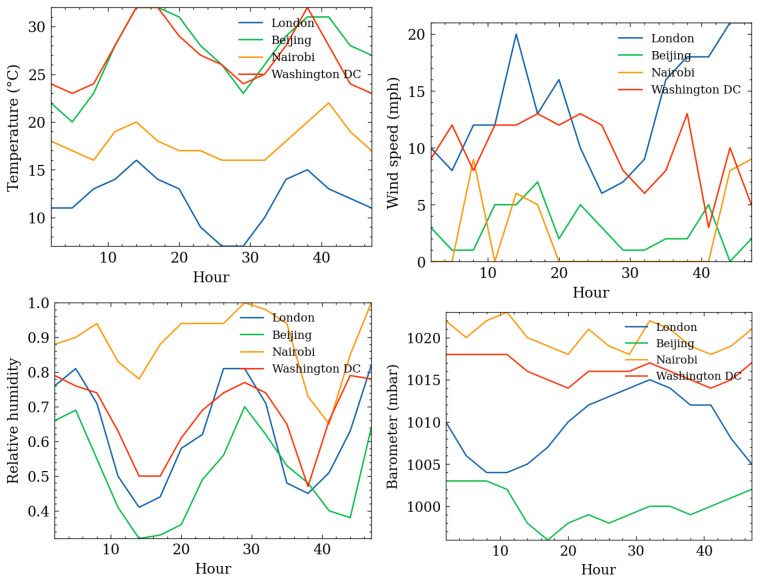
Meteorological changes in four cities on 6 January 2015.

These four cities each represent four different climates: Beijing—warm temperate semi-humid continental monsoon climate; London—humid temperate oceanic climate; Nairobi—subtropical highland climate; Washington DC—humid subtropical climate. At the end of May, the four cities also experienced multi-changeable weather. The selection of these four cities for analysis experiments can better illustrate the universality of the prediction model. Research on wind-speed prediction is of great value. In future work, we will consider the seasonality and diversity of wind-speed variations and extremely severe weather. See Figure 9.

### 4.1. Quality Indicator Discussion

This paper uses the abovementioned QIs to predict wind speed in two directions. Figures of the correlations between wind speed and each QI are shown in Appendix A. According to these figures, the conclusion about the relationships between QIs and predicted wind speed in both directions are summarised below:

1. The correlations between wind speed and each QI are distinct and vary with altitude. For each QI, wind speed is concentrated on a specific value. Take the wind speed of the U component at 875 Hpa as an example. When the divergence is between −0.0003 and 0.0003 s^−1^, the wind speed is likely between −0.8 and 10.4 m/s. With potential vorticity greater than 0 Km^2^kg^−1^s^−1^, the wind speed of the u-component is between −12 and −6.4 m/s.
2. For particular QIs, wind speed can be constrained. For example, when the absolute value of potential vorticity is greater than 0.00001, the wind speed in any direction ranges from −10 to 10 m/s. The range is about two-thirds lower. Suppose the specific cloud ice water content, specific cloud liquid water content, specific rain water content, and specific snow water content gradually increase in value. In that case, the wind speed will converge to zero or a certain value.
3. In addition to special humidity, wind speed will concentrate in a certain area with the rest of the QIs. When diverging around, the probability of wind speed will be small.

It is important to note that these QIs are not independent. Future work will focus on correctly analysing the correlation of these QIs, describing all QIs in a lower dimension, and using this lower dimension as input. This would reduce storage space and training time.

### 4.2. Feature Importance

**Figure 10:**
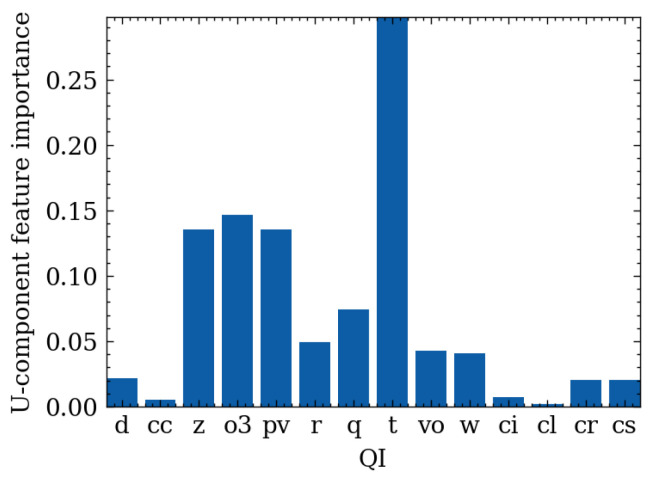
U-component feature importance.

**Figure 11:**
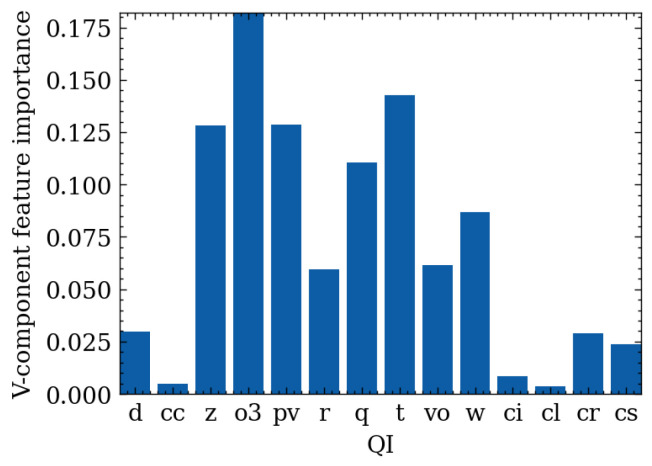
V-component feature importance.

The Permutation Feature Importance (PFI) method was used to calculate the feature importance of the QIs. This method consists of shuffling a random QI value and calculating the performance change in the model. According to Figure 10 and Figure 11, geopotential, ozone mass mixing ratio, potential vorticity, and temperature are the four most important QIs for the wind-speed prediction task. Surprisingly, the relative humidity used primarily in traditional empirical wind-speed prediction models is unimportant in this dataset. However, if the last epochs’ wind-speed is also input into the machine-learning model, experimental results indicate that the importance of the feature exceeds 0.95. These findings indicate that wind speed is highly dependent on time. Therefore, this paper aims to predict wind speed in two directions using those of the past epochs and all QIs. First, wind speed in an hour will be predicted.

### 4.3. Evaluation Metrics

This paper selected Mean Absolute Error (MAE), Root Mean Square Error (RMSE), and Maximum Prediction Error (MAX) as evaluation metrics. MAE and RMSE are the most commonly used metrics for estimating prediction accuracy. MAE is a reflection of prediction errors. In comparison, RMSE measures the deviation between the predicted value and the true value. In addition, considering safety criticality, the maximum error should also be calculated. For example, a smaller maximum error in wind-speed prediction means fewer accident rates for aircraft. These metrics are used in the remainder of the analysis to compare the performance of each model.

(7) $$MAE=\frac{1}{n}\sum_{i=1}^{n}\left|{\hat{x}}_{i}-x_{i}\right|$$

(8) $$RMSE=\sqrt{\frac{1}{n}\sum_{i=1}^{n}{\|{\hat{x}}_{i}-x_{i}\|}_{2}}$$

(9) $$MAX=max\left|{\hat{x}}_{i}-x_{i}\right|$$

where *n* is the number of data points being evaluated. ${\hat{x}}_{i}$ and $x_{i}$ are the predicted and actual values for the *i*-th data point, respectively.

### 4.4. Deep Learning Models

Different ranges of QI values will affect the convergence of the training model. Therefore, all QI values should be normalised to [0,1]. The normalisation formula is

(10) $$x_{i}^{\prime }=\frac{x_{i}-\min x}{\max x-\min x}$$

After QI normalisation, the parameters of classical machine learning and deep learning models should be chosen. In this experiment, the three-order polynomial regression model is chosen. For the random forest model, the maximum depth and number of estimators are 10 and 100, respectively.

**Figure 12:**
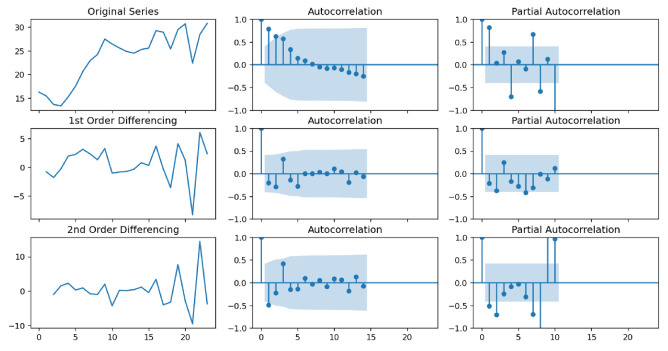
Autocorrelation and Partial Autocorrelation Function plots for U-component wind speed.

**Figure 13:**
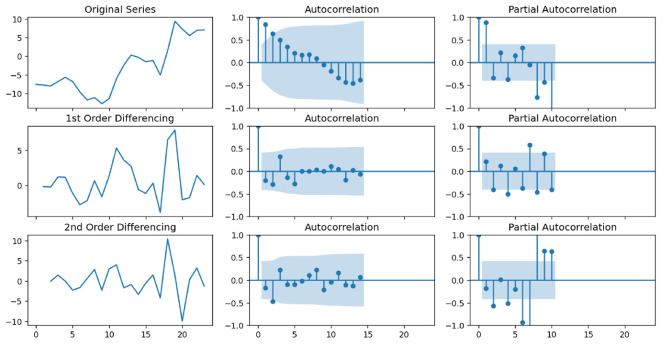
Autocorrelation and Partial Autocorrelation Function plots for V-component wind speed.

Autocorrelation Function (ACF) and Partial Autocorrelation Function (PACF) lollipop plots with varying differential orders have been drawn to determine the ARIMA model parameters (see Figure 12 and Figure 13). The ARIMA model requires a stationary time series as input. Nonstationary time series must be differentiated to become stationary. We selected the differential wind speed of order one as the input based on the results shown in the two plots.

As the AR and MA terms, the values above the confidence interval region (blue) in the PACF and ACF plots should be selected. Consequently, based on the two graphs, we determined the amount of AR to be two and the amount of MA to be zero. In this circumstance, the ARIMA model degenerates into a polynomial regression model, which may be less effective.

For MLP, LSTM, and GRU, three hidden layers are deployed inside. The LSTM or GRU model summary is shown in Table 2. The total training parameters in LSTM and GRU are 129,953 and 98,145, respectively. Because of fewer parameters, the training time of GRU is much faster than that of LSTM. Conversely, the prediction accuracy of GRU will be worse than LSTM’s.

The summary of the CNN-LSTM model is illustrated in Table 3. One two-dimensional CNN structure replaces one LSTM cell for learning hidden features. The total parameters in CNN-LSTM are 640,417, five times more than LSTM.

The Bi-LSTM model is summarised in Table 4. The Leaky Rectified Linear Unit (Leaky ReLU) activation function describes the model non-linearly and prevents vanishing gradient issues. The total parameters in Bi-LSTM are 167,553, which is also less than that of LSTM.

The learning optimiser is Adaptive Moment Estimation (ADAM). By adjusting the learning rate adaptively, the ADAM optimiser can update variables in accordance with the oscillation of the historical gradient and the true historical gradient after filtering the oscillation. Moreover, the loss function is MSE, and the number of epochs for network models is 500.

### 4.5. Wind-Speed Prediction Results

**Figure 14:**
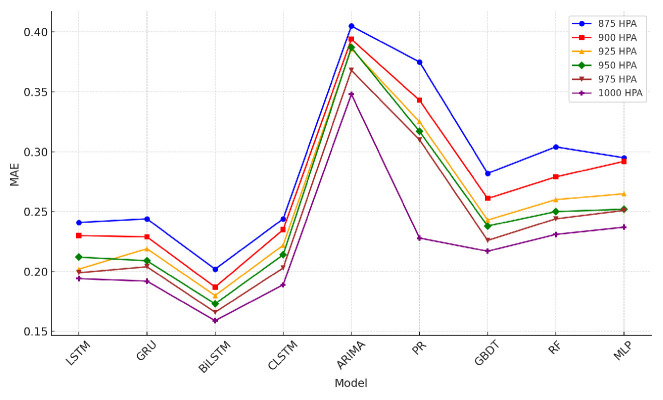
U-direction MAE machine learning results in Beijing.

**Figure 15:**
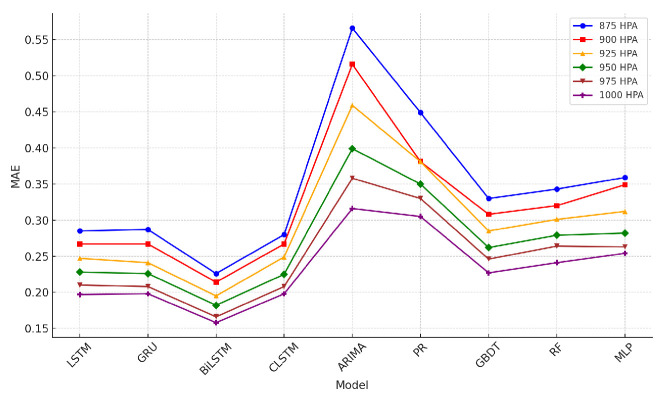
V-direction MAE machine learning results in Beijing.

**Figure 16:**
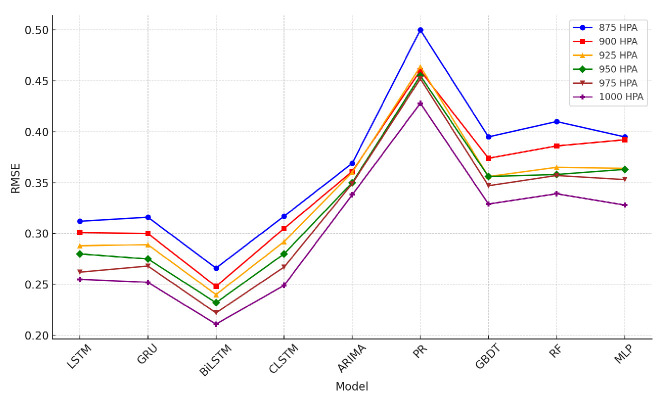
U-direction RMSE machine learning results in Beijing.

**Figure 17:**
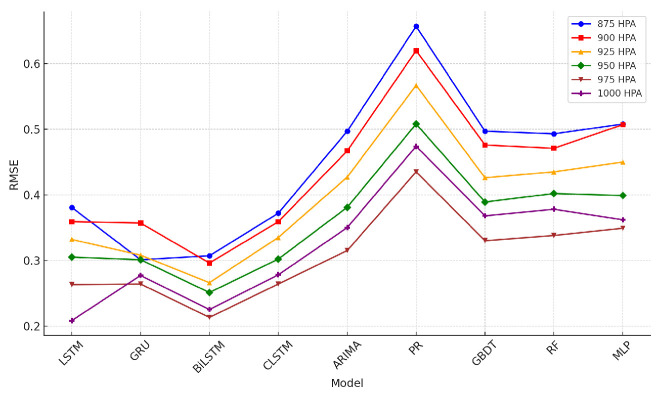
V-direction RMSE machine learning results in Beijing.

**Figure 18:**
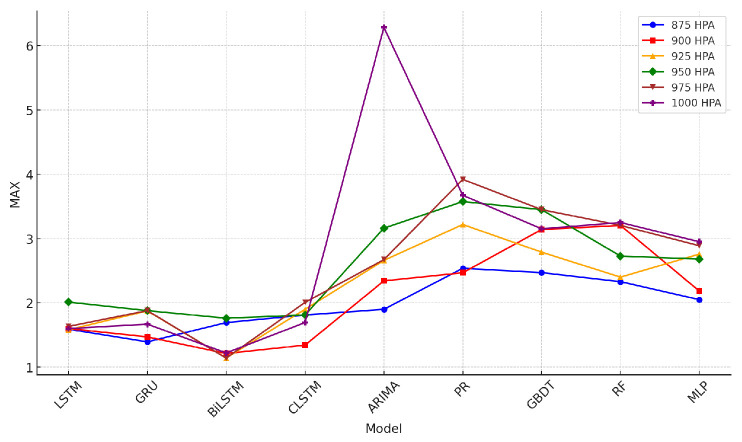
U-direction MAX machine learning results in Beijing.

**Figure 19:**
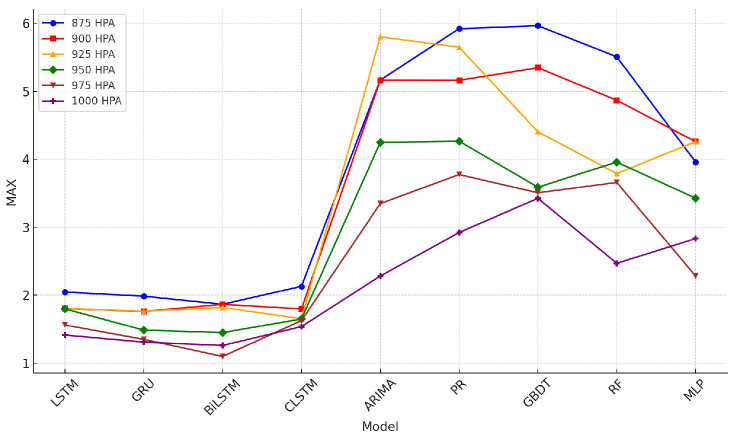
V-direction MAX Machine learning results in Beijing.

**Figure 20:**
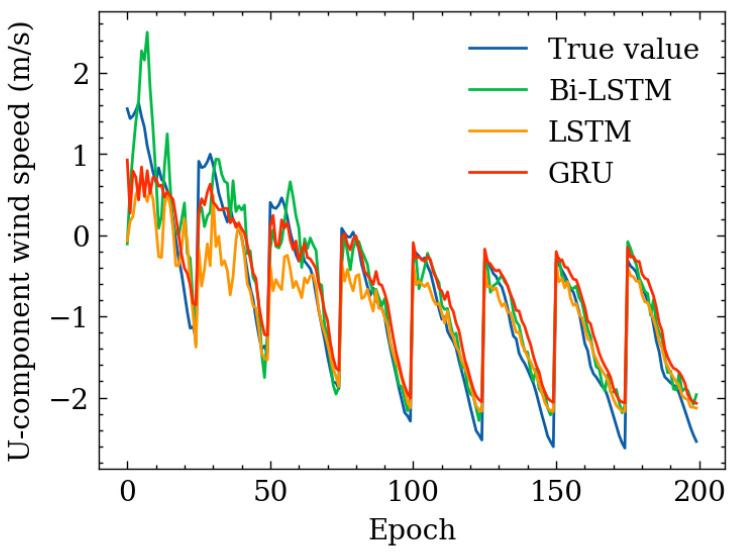
U-component wind-speed prediction result in 1000 hPa, Beijing.

**Figure 21:**
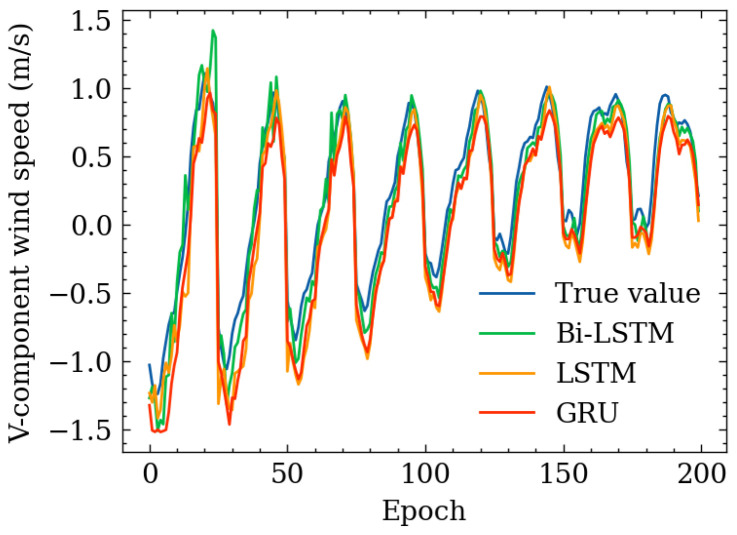
V-component wind-speed prediction result in 1000 hPa, Beijing.

The tables describing horizontal wind-speed prediction results are shown in the Appendix A, where CLSTM, AP, PR, and RF denote CNN-LSTM, Atmospheric Pressure, Polynomial Regression, and Random Forest. The units of MAE, RMSE, and MAX are all m/s. Taking Beijing as an example, the illustrated results are shown in Figure 14, Figure 15, Figure 16, Figure 17, Figure 18 and Figure 19 below. Moreover, the wind-speed prediction results in 1000 hPa are illustrated in Figure 20 and Figure 21.

By comparing the U- and V-component wind-speed prediction results of different traditional point-wise machine learning and temporally correlated deep learning models, the following experimental results can be listed:

1. Traditional machine learning models, including polynomial regression (PR), random forest (RF), Gradient Boosting Decision Tree (GBDT), and MLP, generally showed worse prediction results than deep learning models because they are point- or epoch-wise methods. No temporally correlated information was used for prediction. Not only the prediction performance in MAE and RMSE was worse, but also the maximum prediction errors were always more than twice as large as those of the temporal deep learning models in all cases. This shows that using point-wise models to predict horizontal wind speed will produce large errors, resulting in improper operation and serious accidents. Mission-critical services prohibit this.
2. The ARIMA model also had worse prediction results than deep learning models, and in some altitudes and cities, the results were also worse than point-wise machine learning models, especially GBDT. This is because the amount of MA was zero in this paper. Past predicted errors were not used for parameter tuning. Therefore, the ARIMA model could not analyse the previous prediction error.
3. LSTM, GRU, and CNN-LSTM performed similarly among these four deep-learning models. Normally, CNN-LSTM would be more effective since the hidden features can be extracted through convolution operations. However, the similar performance means that the fourteen QIs were sufficient for the horizontal wind-speed prediction task.
4. In some cases (875 hPA, London, 900 hPA Washington DC and 975 Washington DC), the maximum prediction errors of the CNN-LSTM were better than those of BiLSTM. This illustrated the importance of automatically learning salient features through the CNN layer. GRU’s advantage lies in the training speed, not the prediction accuracy. GRU should be considered when horizontal wind speed needs to be predicted quickly.
5. Bi-LSTM was the best-performing horizontal wind-speed predictor regarding MAE and RMSE. Although Bi-LSTM was occasionally greater than other deep-learning models for the MAX metric, especially CNN-LSTM, forward and backward time-series learning achieves a better prediction effect.

Traditionally, three QIs, atmospheric pressure, relative humidity and temperature, are mainly implemented to predict horizontal wind speed, rather than fourteen. Indeed, these three QIs positively correlate with the horizontal wind speed, also proved in the feature importance analysis mentioned above. However, other QIs are also important, such as the Ozone mass mixing ratio. Therefore, we conducted a comparative experiment to verify the effectiveness of using fourteen features for horizontal wind-speed prediction. Through comparing predicting results with two sets of inputs, including (1) all fourteen QIs. (2) The three QIs mentioned, which are illustrated in Table 5, Table 6, Table 7 and Table 8, (1) always better achieved the MAE and RMSE metrics. Moreover, almost all maximum prediction errors from (1) were smaller than those from (2). There are six cases in which the maximum prediction errors from (1) were greater. This illustrates that future research should focus on the correlation between these fourteen QIs and remove less relevant features to achieve a better effect.

## 5. Conclusions and Future Work

This paper uses fourteen Quality Indicators (QIs) for horizontal wind-speed prediction. Experimental results implementing both traditional point-wise machine learning models, ARIMA, and temporally correlated deep learning models illustrate several results: (i) Deep learning models’ prediction results were better than those of point-wise models. Moreover, Bi-LSTM had the best prediction results in these four deep-learning models. Therefore, forward and backward time-series learning achieves a better prediction effect. (ii) The results when using fourteen QIs as input were much better than those with just atmospheric pressure, relative humidity, and temperature. All metrics are improved to some extent. The fourteen QIs should be further analysed in future work to achieve a better effect. After accurately predicting horizontal wind speed, the results will be used in real-time aircraft operations to reduce deviations from the course due to wind speed, resulting in aircraft delays and accidents.

## Figures and Tables

**Table 1:** Overview of QIs and sensor types.

QI	LIDAR	Radar	Sound Wave	Satellite Signal	Image	Infrared Radiation	Solar Radiation	Inertial Info	Gravity	UV Radiation	Micro-Wave	Electro-Chemical Info	Physical Info
Divergence and velocity	•	•	•	•									
Fraction of cloud cover	•			•	•	•	•						
Geopotential				•				•	•				•
Ozone mass mixing ratio	•				•					•	•	•	
Potential and relative vorticity		•	•	•		•							
Relative and specific humidity	•				•	•					•	•	
Four water content	•	•									•		•
Temperature					•	•						•	

**Table 2:** Model summary of LSTM and GRU.

Layer	Output Shape	Parameters
LSTM (GRU)	(None, 5, 128)	68,096 (51,456)
Dropout	(None, 5, 128)	0
LSTM (GRU)	(None, 5, 64)	49,408 (37,248)
Dropout	(None, 5, 64)	0
LSTM (GRU)	(None, 5, 32)	12,416 (9408)
Dropout	(None, 5, 32)	0
Dense	(None, 5, 1)	33 (33)

**Table 3:** Model summary of CNN-LSTM.

Layer	Output Shape	Parameters
Reshape	(None, 5, 16, 1)	0
Conv2D	(None, 5, 16, 64)	640
MaxPooling	(None, 5, 16, 64)	0
Dropout	(None, 5, 16, 64)	0
Reshape	(None, 5, 128)	0
LSTM	(None, 5, 128)	590,336
Dropout	(None, 5, 128)	0
LSTM	(None, 5, 64)	49,408
Dense	(None, 5, 128)	33

**Table 4:** Model summary of Bi-LSTM.

Layer	Output Shape	Parameters
Bidirectional	(None, 256)	148,480
Dense	(None, 64)	16,448
Leaky ReLU	(None, 64)	0
Dense	(None, 43)	2080
Leaky ReLU	(None, 43)	0
Dense	(None, 16)	528
Leaky ReLU	(None, 16)	0
Dense	(None, 1)	17
Leaky ReLU	(None, 1)	0

**Table 5:** Beijing’s BiLSTM results comparison with (1) all QIs and (2) atmospheric pressure, relative humidity and temperature only.

Input	Direction	Altitude	MAE	RMSE	MAX
(1)	u	875	0.202	0.266	1.693
(2)	u	875	0.207	0.274	2.049
(1)	v	875	0.226	0.307	1.495
(2)	v	875	0.235	0.322	2.022
(1)	u	900	0.187	0.248	1.401
(2)	u	900	0.197	0.261	1.433
(1)	v	900	0.214	0.296	1.866
(2)	v	900	0.217	0.300	2.067
(1)	u	925	0.180	0.240	1.496
(2)	u	925	0.186	0.249	1.687
(1)	v	925	0.195	0.266	1.815
(2)	v	925	0.203	0.281	1.844
(1)	u	950	0.173	0.232	1.761
(2)	u	950	0.174	0.232	1.597
(1)	v	950	0.182	0.251	1.449
(2)	v	950	0.181	0.248	1.611
(1)	u	975	0.166	0.222	1.141
(2)	u	975	0.169	0.226	1.400
(1)	v	975	0.166	0.225	1.098
(2)	v	975	0.167	0.227	1.356
(1)	u	1000	0.159	0.211	1.213
(2)	u	1000	0.167	0.223	1.099
(1)	v	1000	0.158	0.213	1.260
(2)	v	1000	0.161	0.219	1.338

**Table 6:** London’s BiLSTM results comparison with (1) all QIs and (2) atmospheric pressure, relative humidity and temperature only.

Input	Direction	Altitude	MAE	RMSE	MAX
(1)	u	875	0.282	0.365	1.904
(2)	u	875	0.307	0.397	2.139
(1)	v	875	0.297	0.385	2.476
(2)	v	875	0.358	0.472	3.643
(1)	u	900	0.272	0.350	1.854
(2)	u	900	0.309	0.401	1.874
(1)	v	900	0.309	0.402	3.479
(2)	v	900	0.373	0.495	4.430
(1)	u	925	0.278	0.360	2.061
(2)	u	925	0.312	0.408	2.871
(1)	v	925	0.317	0.413	2.259
(2)	v	925	0.395	0.536	4.879
(1)	u	950	0.287	0.371	1.978
(2)	u	950	0.335	0.436	2.977
(1)	v	950	0.311	0.408	2.381
(2)	v	950	0.407	0.561	6.523
(1)	u	975	0.295	0.381	1.915
(2)	u	975	0.351	0.466	2.900
(1)	v	975	0.317	0.416	2.475
(2)	v	975	0.410	0.578	8.291
(1)	u	1000	0.285	0.372	1.936
(2)	u	1000	0.320	0.424	2.398
(1)	v	1000	0.286	0.379	2.780
(2)	v	1000	0.323	0.436	4.483

**Table 7:** Nairobi’s BiLSTM results comparison with (1) all QIs and (2) atmospheric pressure, relative humidity and temperature only.

Input	Direction	Altitude	MAE	RMSE	MAX
(1)	u	875	0.311	0.423	2.749
(2)	u	875	0.318	0.433	2.898
(1)	v	875	0.269	0.363	2.516
(2)	v	875	0.283	0.381	2.493
(1)	u	900	0.276	0.376	2.915
(2)	u	900	0.293	0.396	3.220
(1)	v	900	0.244	0.326	2.059
(2)	v	900	0.266	0.357	2.181
(1)	u	925	0.272	0.365	2.613
(2)	u	925	0.280	0.379	2.960
(1)	v	925	0.241	0.322	2.086
(2)	v	925	0.262	0.353	2.013
(1)	u	950	0.265	0.361	2.692
(2)	u	950	0.274	0.373	2.841
(1)	v	950	0.237	0.315	1.869
(2)	v	950	0.254	0.344	2.930
(1)	u	975	0.260	0.351	2.301
(2)	u	975	0.271	0.370	2.635
(1)	v	975	0.232	0.309	1.706
(2)	v	975	0.244	0.330	1.928
(1)	u	1000	0.249	0.336	2.147
(2)	u	1000	0.264	0.357	2.345
(1)	v	1000	0.223	0.299	1.816
(2)	v	1000	0.234	0.314	1.706

**Table 8:** Washington DC’s BiLSTM results comparison with (1) all QIs and (2) atmospheric pressure, relative humidity and temperature only.

Input	Direction	Altitude	MAE	RMSE	MAX
(1)	u	875	0.386	0.505	2.737
(2)	u	875	0.432	0.571	2.594
(1)	v	875	0.347	0.452	3.148
(2)	v	875	0.365	0.478	3.084
(1)	u	900	0.351	0.457	2.479
(2)	u	900	0.390	0.511	2.719
(1)	v	900	0.323	0.422	2.650
(2)	v	900	0.359	0.469	2.898
(1)	u	925	0.337	0.439	2.243
(2)	u	925	0.369	0.483	2.221
(1)	v	925	0.300	0.392	2.020
(2)	v	925	0.315	0.411	2.659
(1)	u	950	0.317	0.417	2.859
(2)	u	950	0.327	0.431	2.876
(1)	v	950	0.287	0.375	2.310
(2)	v	950	0.290	0.379	2.438
(1)	u	975	0.290	0.383	3.175
(2)	u	975	0.302	0.400	3.213
(1)	v	975	0.255	0.337	2.297
(2)	v	975	0.258	0.342	2.240
(1)	u	1000	0.258	0.345	2.279
(2)	u	1000	0.273	0.363	2.416
(1)	v	1000	0.219	0.294	2.260
(2)	v	1000	0.226	0.303	2.062

## Data Availability

Data used in this paper are from ECMWF Reanalysis v5 (ERA5).

## Appendix A

See https://github.com/woollling-li/Paper-Appendix accessed on 1 January 2022.
